# Supplementary material for: Positive feedback regulation of frizzled-7 expression robustly shapes a steep Wnt gradient in Xenopus heart development, together with sFRP1 and heparan sulfate
Source: eLife. 2022 Aug 10;11:e73818. doi: 10.7554/eLife.73818 (PMC9363125; doi:10.7554/eLife.73818)
Supplement: Supplementary file 1. [file elife-73818-supp1.docx]

**Supplementary File 1. RNA probe synthesis for *in situ* hybridization.**

| Gene | Restriction  enzyme | Transcription  Enzyme | References |
| --- | --- | --- | --- |
| *fzd7* (Full-length) | *Hind*III | T3 | This paper |
| *fzd7* (intracellular domain) | *Ava*I | SP6 | This paper |
| *gata5* | *Spe*I | T7 | This paper |
| *myl2* | *Bam*HI | T7 | (Evans et al., 1995) |
| *tnni3* | *Not*I | T7 | (Drysdale et al., 1994) |
