# Supplementary material for: Positive feedback regulation of frizzled-7 expression robustly shapes a steep Wnt gradient in Xenopus heart development, together with sFRP1 and heparan sulfate
Source: eLife. 2022 Aug 10;11:e73818. doi: 10.7554/eLife.73818 (PMC9363125; doi:10.7554/eLife.73818)
Supplement: Supplementary file 2. [file elife-73818-supp2.docx]

**Supplementary File 2. PCR primers used in this study.**

For mRNA synthesis:

|  | Sequence |
| --- | --- |
| Fzd7CRD-Fcr | GATCTCCATGTCCTCTACAGTCTCGCT |
| Fzd7CRD-Rcr | TCTCGTCCAGCCCGTTGGCTTTGCC |
| Tcf-F | AGGTAAAGAATGTAACTGTATTC |
| Tcf-R | GGAGGACATGGAGATCTGCG |
| IgG-F | ACGGGCTGGACGAGACC |
| IgG-R | TTACATTCTTTACCTTGAAGAGTGT |
| cgBamHI-nosigxlWnt6S-F | CGggatccCTTTGGTGGGCAGTTGGCA |
| gcXbaI-Wnt6S-R | GCtctagaTCATAGACACACACTCAGTTCTT |
| cgBamHI-xlWnt6S-F | CGggatccaccATGTTGCCCCTGTCCAGAT |
| gcXbaI-nostopWnt6S-R | GCtctagaTAGACACACACTCAGTTCTTTTTT |
| cgBamHI-nosigxlSfrp1L-F | CGggatccTCCCAGGTTCCCCAAGC |
| gcXbaI-Sfrp1-R | GCtctagaTTAATGGGGGGCTGTGAGC |
| extraNotI-kzk-Ndst1-F | ATAAGAATgcggccgcTCCACCATGAGCTTGTCCC |
| cgMluI-Ndst1-R | CGacgcgtTTACCTAGTGTTCTGAAGCTC |

For RNA probe synthesis:

| Fzd7Cterm-F | GTATCTGGGCGATCCTGTG |
| --- | --- |
| Fzd7Cterm-R | ACCGCAGTCTCCCCTTTG |
| Gata5-F | CTCCGATTAGATCGAAACAAG |
| Gata5-R | TAGTCAGACAGGGAACACATT |
